# Coupling Coordination Analysis of Medical Service Capacity, Residents’ Health Level and Regional Economic Development in China

**DOI:** 10.3390/healthcare14152241

**Published:** 2026-07-23

**Authors:** Xiaomin Xu, Xiangyang Gong

**Affiliations:** Department of Radiology, Cancer Center, Zhejiang Provincial People’s Hospital, Affiliated People’s Hospital, Hangzhou Medical College, Hangzhou 310014, China; 130232023259@hmc.edu.cn

**Keywords:** medical service capacity, residents’ health level, regional economic development, coupling coordination degree, spatial pattern

## Abstract

**Background**: Synergistic development of the coupled and coordinated relationship of medical service capacity, residents’ health level, and regional economic development (MRR) is crucial for realizing the “Healthy China 2030” strategy. This study aims to analyze the spatiotemporal features of the coupling coordination degree (CCD) of MRR. **Methods**: Based on panel data from 2012 to 2022, we constructed the MRR evaluation system. The entropy method was adopted to calculate the comprehensive index of each subsystem, and the coupling coordination degree model (CCDM) was used to evaluate the CCD of MRR during the study period. Furthermore, we applied exploratory spatial data analysis, the Dagum Gini coefficient decomposition, extreme gradient boosting (XGBoost) and Shapley Additive Explanations (SHAP) to explore spatiotemporal dynamic characteristics and factors associated with CCD. **Results:** (1) Throughout the study period, the overall value of the MRR composite system increased from 0.3798 to 0.4097, with an average annual growth rate of 0.54%. (2) CCD remained generally low, dominated by the basic coordination type. Both global and local spatial autocorrelation analyses revealed significant differentiated spatial clusters of high and low CCD, with the overall spatial distribution fitting a “high in the east, low in the west” pattern. Inter-regional gaps are the main reason for the overall differences. (3) In the full sample, Population density (PD)was the dominant external predictive contributor, with a mean absolute SHAP value of 0.028, followed by Internet broadband access ports (IBAP)and Government health expenditure (GHE). The key predictive factors differed among regions. **Conclusions:** This study clarifies spatiotemporal evolution and predictive contributors of CCD. These findings provide valuable insights for decision-makers to promote coordinated MRR development.

## 1. Introduction

With the continuous improvement of global health governance and rapid socioeconomic advancement, health has gained growing strategic prominence as a fundamental pillar of socioeconomic development and national well-being. Against this backdrop, the interactive coupling among medical service capacity, residents’ health level, and regional economic development (MRR) has become a critical research issue in public health and economic governance. Since the issuance of the Healthy China 2030 Planning Outline, China has upheld a people-centered health philosophy, driving the health sector to transform from disease-oriented treatment to comprehensive whole-population health promotion. Clarifying the theoretical mechanisms and practical synergies among the three dimensions is essential for optimizing health resource allocation and achieving coordinated regional development.

From a theoretical perspective, their interactive relationships operate through multiple clear pathways. First, guided by public goods theory and health economics, economic development acts as the material foundation for medical service capacity upgrading. Sufficient fiscal investment and economic endowments provide basic resources for medical infrastructure construction, talent cultivation and daily operation of health systems; nevertheless, economic input only serves as a core rather than a decisive factor, and its effectiveness is constrained by institutional arrangements, management modes and resource allocation efficiency [1]. Second, based on human capital theory, a sound medical service system is a core guarantee for maintaining and improving population health. Multidimensional medical capacity, including routine service, emergency response and public health intervention, reduces disease burden, extends life expectancy and optimizes public health status [2]. Third, residents’ health level further feeds back into economic growth: healthy human capital improves labor productivity, curbs the economic losses caused by diseases and public health crises, and creates favorable conditions for sustainable regional economic development [3].

Extensive research has explored bilateral relationships in medical service capacity, residents’ health level, and regional economic development. First, existing literature has confirmed that medical service capacity is closely associated with residents’ health level, with healthcare accessibility, service quality and resource distribution playing essential roles. Temesgen [4] conducted a study in Addis Ababa, Ethiopia and found that healthcare accessibility, travel time, and waiting time significantly influence the utilization of maternal health services, which further directly affects maternal health outcomes. Noviyani [5] carried out a qualitative study in Palembang, Indonesia and highlighted that patients’ perceived service quality, including staff professionalism, medical facilities, and patient-provider communication, is a key determinant of patient satisfaction and health-seeking behaviors. Second, the relationship between medical service capacity and regional economic development represents a complex, two-way interaction. Qin [6] conducted a nationwide study covering 290 cities in China and demonstrated that uneven economic development significantly drives the inequitable geographic distribution of healthcare resources, revealing that economically advantaged regions tend to attract and retain more physicians and hospital beds, thereby strengthening regional medical service capacity. Guo [7] further confirmed that targeted healthcare reforms can effectively mitigate medical resource misallocation by increasing government health investment and optimizing health insurance structures, highlighting the critical role of economic-supporting policies in enhancing the equity and efficiency of medical service provision. Third, extensive research has verified the bidirectional relationship between residents’ health level and regional economic development. Portnoy [8] conducted a modeling study across 105 low- and middle-income countries (LMICs) worldwide and demonstrated that improving population health through novel tuberculosis vaccine introduction can reduce disease-related poverty, expand the labor supply, and further stimulate national economic growth. Mayer [9] focused on 18 Latin American countries and further revealed that better population health continuously promotes long-term regional economic growth by extending workers’ active labor years and enhancing labor productivity, highlighting that health serves as a fundamental form of human capital underlying sustainable economic development.

Despite these contributions, two critical gaps remain in current literature. First, existing studies worldwide have primarily focused on either bilateral relationships or single-country contexts, with limited systematic investigation into the tripartite, synergistic interactions, especially in large developing economies like China. Most studies adopt a unidirectional or dyadic perspective, overlooking the complex, mutually reinforcing interactions among MRR composite systems [10]. To address this, we introduce the concept of coupling coordination [11] and apply the coupling coordination degree model (CCDM) to quantify the synergy among the MRR composite systems. Second, prior studies predominantly use linear models, which may fail to capture the nonlinear and high-dimensional relationships inherent in such complex systems. To overcome this limitation, we employ an XGBoost (version 2.1.3) algorithm. In contrast to traditional linear regressions, this non-parametric approach avoids subjective functional form assumptions, accommodates high-dimensional and complex data structures, and can effectively handle outliers, multicollinearity, and overfitting [12].

This study draws on panel data from 31 provinces in China and employs a variety of research methodologies, including the entropy method, the coupling coordination degree model (CCDM), exploratory spatial data analysis (ESDA), the Dagum Gini coefficient, the Extreme Gradient Boosting (XGBoost) and Shapley Additive Explanations (SHAP). The aim is to explore the coupling coordination degree (CCD) and predictive contributors among MRR from 2012 to 2022. 

## 2. Materials and Methods

### 2.1. Study Region

**Figure 1:**
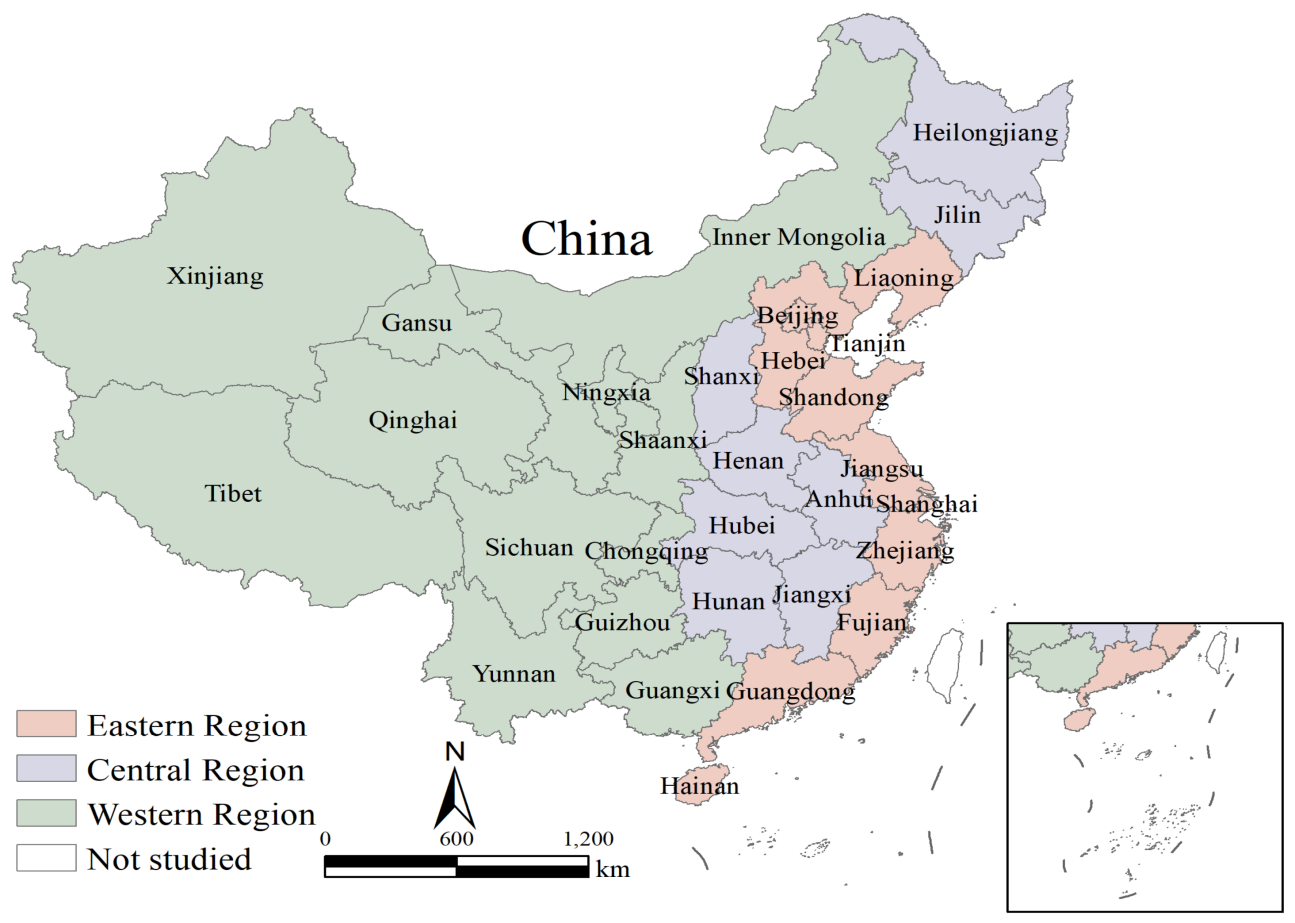
Research Division.

This study focuses on 31 provinces in mainland China, excluding the Hong Kong Special Administrative Region, the Macao Special Administrative Region, and Taiwan Province. The 31 provinces are categorized into three administrative regions: the Eastern Region, which includes Beijing, Tianjin, Hebei, Liaoning, Shanghai, Jiangsu, Zhejiang, Fujian, Shandong, Guangdong, and Hainan; the Central Region, which includes Henan, Hubei, Hunan, Anhui, Jiangxi, Shanxi, Jilin and Heilongjiang; and the Western Region, which includes Inner Mongolia, Guangxi, Chongqing, Sichuan, Guizhou, Yunnan, Tibet, Shaanxi, Gansu, Qinghai, Ningxia, and Xinjiang. The geographical locations of the 3 administrative regions are shown in Figure 1.

### 2.2. Data Sources

Data on provincial medical service capacity, residents’ health levels, and regional economic development from 2012 to 2022, as well as external predictors associated with MRR, are sourced from the China Statistical Yearbook, China Health Statistics Yearbook, China Health and Family Planning Statistical Yearbook, China Population and Employment Statistical Yearbook, China Social Statistical Yearbook, China Environmental Statistical Yearbook, and other relevant yearbooks, All data were accessed via the CNKI China Economic and Social Big Data Research Platform. For missing data points in certain years, linear interpolation was applied to fill the gaps, ensuring data completeness and the continuity of the time series analysis. The compound annual growth rate (CAGR) was used to calculate the average annual growth rate.

### 2.3. Data Preprocessing

Due to the presence of positive and negative indicators, as well as differences in measurement units and orders of magnitude among various indicators, standardization is required to enhance the comparability of different indicators. The standardization calculations are as follows [13]:

(1) $${\;Y}_{ij}=\frac{X_{ij}-X_{ijmin}}{X_{ijmax}-X_{ijmin}}\left(i\;=1,2,\cdots ,n;j=1,2\cdots ,m\right)(positive\;index)$$

(2) $$Y_{ij}=\frac{X_{ijmax}-X_{ij}}{X_{ijmax}-X_{ijmin}}\left(i=1,2,\cdots ,n;j=1,2\cdots ,m\right)\;\;(negative\;index)$$

Here, $X_{ij}$ represents the original value of the indicator; $X_{ijmin}$ denotes the minimum value of the j  indicator for the  i evaluation object; and ${\;X}_{ijmax}$ refers to the maximum value of the j indicator for the i evaluation object. Assuming the number of evaluation objects and evaluation indicators are m and *n*, respectively, then $X_{ij}$ ≥ 0, with 0 ≤ i ≤ m, 0 ≤ j ≤ n.

### 2.4. Construction of the Indicator System

The indicator system for the MRR composite system is developed based on previous studies, adhering to the principles of data availability, indicator representativeness and systematic relevance. We adopt a hierarchical indicator framework for three subsystems, with all indicators categorized as positive or negative. A higher value of positive indicators represents better subsystem development, whereas a higher value of negative indicators indicates worse performance.

In the medical service capacity subsystem, we follow the classic three-dimensional framework of medical input, output and efficiency for indicator selection. Number of medical institutions per 1000 people, number of beds per 1000 people, and number of health personnel per 1000 people (C1–C3) reflect basic medical resource supply [14]. Number of outpatient visits, number of inpatient visits, and average daily number of patient visits per doctor (C4–C6) measure medical service output [15,16,17]. Average length of hospital stays and bed utilization rate (C7–C8) characterize operational efficiency and service quality [17]. This set of indicators fully covers resource endowment, service scale and operational performance.

In the residents’ health level subsystem, indicators are selected in accordance with the official standards of the Healthy China 2030 Planning Outline and data accessibility. Incidence rate of Class A and B statutory infectious diseases and average life expectancy (C9–C10) reflect overall population health and public health governance effects [18]. Perinatal mortality rate and maternal mortality rate (C11–C12) are used to evaluate maternal and child health [19].

In the regional economic development subsystem, we follow the classic three-dimensional framework of economic structure, economic vitality, and economic scale for indicator selection. The share of primary industry in GDP, the share of secondary industry in GDP, and the share of tertiary industry in GDP (C13–C15) reflect regional industrial structure and layout [20]. GDP growth rate, foreign trade dependence, and consumption rate (C16–C18) measure regional economic vitality, where the GDP growth rate reflects economic growth momentum, foreign trade dependence measures reliance on overseas markets, and the consumption rate indicates the contribution of domestic demand to economic growth [21]. GDP, fixed-asset investment, and government budget revenue (C19–C21) characterize regional economic scale and comprehensive economic strength [22]. This set of indicators fully covers industrial configuration, growth momentum, and aggregate economic performance. The evaluation indicators within each subsystem are interconnected, forming an interactive and interrelated framework (Table 1).

### 2.5. Method

#### 2.5.1. The Entropy Method

The entropy method is an objective weighting method that determines indicator weights based on the amount of information each indicator provides. Its key advantage lies in ensuring the objectivity and scientific rigor of the weighting process, thereby improving the credibility and stability of evaluation results [23]. The calculation steps are as follows:

(3) $$P_{ij}=\frac{X_{ij}}{\sum_{i\;=1}^{m}\;X_{ij}}$$

(4) $$e_{j}=-\frac{1}{{ln}_{m}}\sum_{i=1}^{m}\;P_{ij}lnP_{ij}$$

(5) $${\;\;\;\;g}_{j}=1-e_{j}$$

(6) $$w_{j}=\frac{g_{j}}{\sum_{j=1}^{n}\;g_{j}}$$

Here, $P_{ij}$ represents the proportion of the j indicator in the i year; $e_{j}$, $g_{j}$ and $w_{j}$ respectively denote the entropy value, difference coefficient, and weight of the j indicator. Based on the above calculation formulas, the specific weights of each indicator in each subsystem are obtained, as shown in Table 1. To test the robustness of the entropy weight method, the coefficient variation method was adopted to recalculate the weights of the three subsystems: Medical service capacity, Residents’ health level, and Regional economic development. The results were then compared with those obtained from the original entropy weight method. Under the two weighting schemes, both the Pearson correlation coefficient and the Spearman rank correlation coefficient of the weights derived from the two methods were 0.992. This indicates that the weight results from the entropy weight method are insensitive to the choice of weighting method, exhibit high robustness, and the derived weight distribution is reliable.

#### 2.5.2. The Coupling Coordination Degree Model (CCDM)

Although the coupling degree reflects the tightness of interaction between two or more systems in regional comparative studies, a single indicator is insufficient to fully reflect the synergistic effect arising from spatial heterogeneity and dynamic evolution. Therefore, CCDM is introduced as a quantitative indicator to measure the coordination status between systems, revealing both the interaction intensity and the overall development status [24]. The calculation steps are as follows:

(7) $$CD\;=\;\frac{\left(MSC\;\times RHL\;\times RED\right)}{\eta_{w}MSC\;+\eta_{n}RHL\;+\eta_{r}RED}$$

(8) $$CCD=\sqrt{CD\times \left(\eta_{w}MSC+\eta_{n}RHL+\eta_{r}RED\right)}$$

Here, the CD represents the coupling degree among MRR composite systems. CCD denotes the coupling coordination degree among MRR composite systems, while α, β, and δ are undetermined coefficients. In this study, it is considered that MRR composite systems are of equal importance in the evaluation of coupled and coordinated development. Therefore, the values of the undetermined coefficients are set as α = β = γ = 1/3 [25].

To further test the robustness of the equal-weight assumption, this study conducted a sensitivity analysis. Keeping the subsystem composite scores unchanged, we tried two alternative weighting schemes (Table 2). Scheme 1: Considering the core role of residents’ health level as the fundamental goal of medical and economic development, a slightly higher weight was assigned to this dimension: α = 0.3, β = 0.4, γ = 0.3 (Table S1). Scheme 2: Given the critical role of medical service capacity as the direct carrier of health service delivery, a slightly higher weight was assigned to this dimension: α = 0.4, β = 0.25, γ = 0.35 (Table S2). The results show that the correlation coefficients between the CCD under the two alternative schemes and that under the original equal-weight scheme both exceeded 0.95; this indicates that our CCD estimates are highly robust to weight specification, and the equal-weight assumption is acceptable.

Utilizing the research findings of related scholars [26], the numerical values of the CCD are divided into ranking criteria (Table 3).

#### 2.5.3. Exploratory Spatial Data Analysis (ESDA)

ESDA is a statistical method used to evaluate the distribution characteristics of spatial data and their interrelationships. Its core premise is that data from spatially adjacent or nearby locations tend to exhibit a certain level of dependence or similarity, which weakens or disappears as the distance between locations increases. Spatial autocorrelation can be divided into two types: global spatial autocorrelation and local spatial autocorrelation, which are used to describe the spatial distribution pattern of the entire study area and the spatial heterogeneity of local areas, respectively. The global Moran’s Index is used to test the existence of spatial autocorrelation, and its calculation formula is as follows [27]:

(9) $$\;\;\;I\;=\frac{n\sum_{i\;=1}^{n}\;\sum_{j\;=1}^{n}\;W_{ij}(X_{i}-\bar{X})\left(X_{j}-\bar{X}\right)}{\sum_{i\;=1}^{n}\;\sum_{j\;=1}^{n}\;W_{ij}\sum_{i\;=1}^{n}\;{\left(X_{i}-\bar{X}\right)}^{2}}$$

Here, $X_{i}$ and $X_{j}$ represent the observed values of the i and j provinces, respectively; $\bar{X}$ is the average value of all sample observations. n is the number of provinces; $W_{ij}$ is a weight indicating the adjacency degree of spatial objects (if region i and region j are adjacent, $W_{ij}$ = 1); otherwise, $W_{ij}$ = 0; the value range of I is from −1 to 1.

However, the global Moran’s index can only explain the average level of similarity in the entire study area. To capture localized effects, local Moran’s Index is used to analyze the spatial clustering of high or low variable values. This index explicitly analyzes local spatial correlation and classifies it into four types: high-high, low-low, high-low, and low-high.

#### 2.5.4. Dagum Gini Coefficient

Compared with the traditional Gini coefficient, the Dagum Gini coefficient and its related decomposition method can not only capture the overall disparities within the sample but also reveal the differences within sub-groups and between sub-groups. This study uses this method to analyze the regional differences of CCD at the overall level and within the three major regions of the east, central and west. The calculation steps are as follows [28]:

(10) $$\;G\;=\sum_{j\;=1}^{k}\;\sum_{h\;=1}^{k}\;\sum_{i\;=1}^{n_{j}}\;\sum_{r\;=1}^{n_{h}}\;\left|y_{ji}-y_{hr}\right|/2n^{2}\bar{y}$$

Here, n represents the number of China’s provincial administrative region; k  represents the number of regional divisions; $n_{j}$ and $n_{h}$ represent the number of provinces included in region j and region h respectively. y stands for the average value of CCD across all regions; $y_{ji}$ denotes the CCD level of province i in region j; $y_{hr}$ indicates the CCD level of province r in region  h. The calculation steps are as follows [29]:

(11) $$G=G_{w}+G_{nb}+G_{i}$$

$G_{w}$ represents the contribution of inter-regional variance, $G_{nb}$ denotes the contribution of intra-regional variance, and $G_{i}$ stands for the contribution of super-variable density.

#### 2.5.5. Extreme Gradient Boosting Model

All analyses were conducted in Python 3.12 with a fixed random seed of 42. The external-factor analysis used a balanced province-year panel covering 31 mainland Chinese provinces from 2012 to 2022, yielding 341 observations. Extreme Gradient Boosting (XGBoost), developed by Chen and Guestrin [30], was used as a predictive modelling tool to capture nonlinear associations between external factors and CCD. To reduce overfitting risk in the relatively small panel dataset, model complexity was constrained by shallow trees, a low learning rate, subsampling, column subsampling, and regularization. The main hyperparameters were set as follows: 120 trees, maximum tree depth of 2, learning rate of 0.035, subsample ratio of 0.8, column subsample ratio of 0.85, L1 regularization of 0.1, and L2 regularization of 2.0. Model performance was evaluated using repeated five-fold cross-validation with 20 repeats. The reported metrics were the coefficient of determination (R^2^), root mean squared error (RMSE) and mean absolute error (MAE). Full-sample and region-specific models were estimated separately for overall, eastern, central, and western samples. Moreover, compared with classic machine learning approaches, algorithmic improvements and parallelization enable it to deliver substantially higher efficiency during training and prediction [31].

#### 2.5.6. Shapley Additive Explanation Interpretation Framework

Although XGBoost can capture nonlinear predictive associations between external factors and CCD, its fitted structure is not directly interpretable. Shapley additive explanations (SHAP), proposed by Lundberg and Lee in 2017 [32] were therefore used to interpret the contribution of each predictor to the model output. Based on Shapley values from cooperative game theory, SHAP decomposes each prediction into additive feature contributions and provides a consistent measure of model-based importance. In this study, absolute SHAP values were used to rank the external predictors, and SHAP distributions were used to describe heterogeneity in their predictive contributions. Because the analysis is based on observational panel data and a predictive machine-learning framework, SHAP values were interpreted as associations with model predictions rather than causal effects.

## 3. Results

### 3.1. Analysis of the Subsystem Development Level

**Figure 2:**
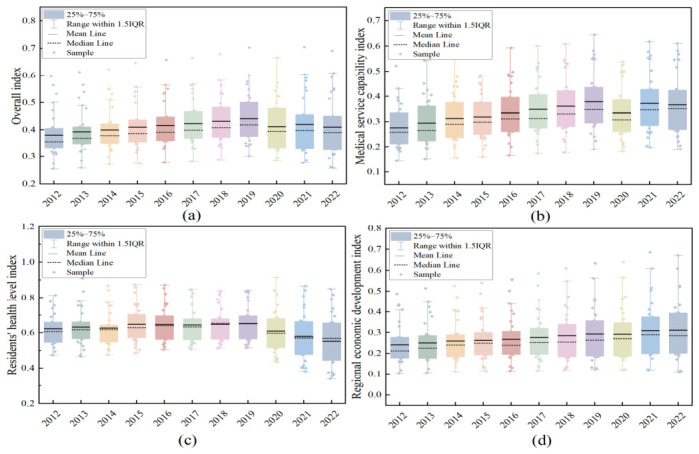
Sub-system composite index. (**a**) Overall index; (**b**) Medical service capacity index; (**c**) Residents’ health level index; (**d**) Regional economic development index.

In this study, the entropy method was adopted to calculate the comprehensive level of each subsystem (Figure 2a). The overall value of the MRR composite system increased from 0.380 to 0.401, with an average annual growth rate of 0.54%. Further analysis of individual subsystems revealed that the medical service capacity index rose from 0.276 to 0.366, corresponding to an average annual growth rate of 2.86%. The regional economic development index climbed from 0.241 to 0.310, with an average annual growth rate of 2.55%. In contrast, the residents’ health level index, which remained the highest among the three subsystems throughout the period, declined from 0.623 to 0.553, corresponding to an average annual growth rate of −1.18% (Figure 2b,d).

### 3.2. Temporal Characteristics and Spatial Patterns of the CCD

#### 3.2.1. Temporal Dimension Analysis

**Figure 3:**
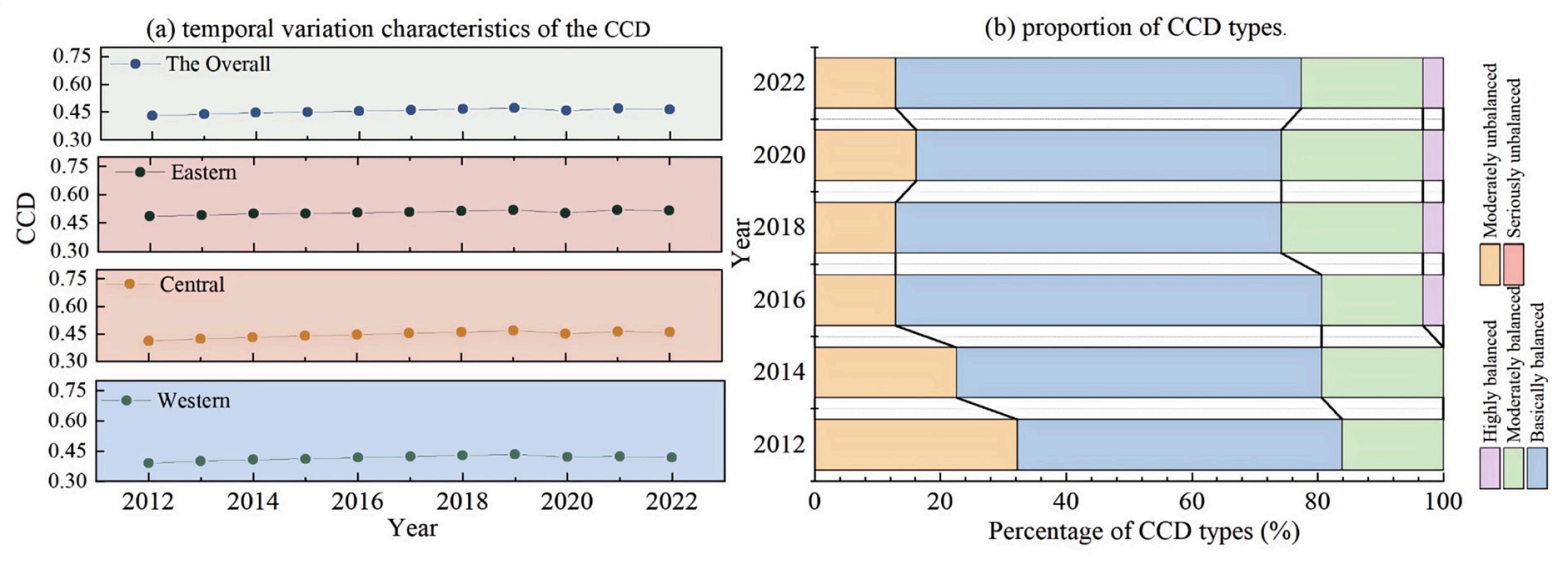
Temporal trends of CCD. (**a**) temporal variation characteristics of the CCD; (**b**) proportion of CCD types.

From a temporal change perspective (Figure 3a), the overall average CCD values across China experienced an upward trend during the study period, rising from 0.429 to 0.463 with an average annual growth rate of 0.77%. Regionally, the mean values increased from 0.510 to 0.545 in the eastern region, 0.392 to 0.418 in the western region, and 0.440 to 0.479 in the central region. While the eastern region always had the highest absolute level, regional growth rates showed evident convergence, ranked as central (0.85%), eastern (0.67%) and western (0.64%).

According to the changes in the proportion of CCD types (Figure 3b), in the initial stage (2012), the majority of counties were in the state of moderate unbalance and basic coordination, collectively accounting for approximately 83.9% of the total, while high-level coordination was entirely absent, indicating that the regional system was still in a transitional phase. During the subsequent adjustment period (2014–2020), the proportion of counties with basic coordination remained relatively high, peaking at 67.7% in 2016, whereas moderate unbalance dropped markedly from 22.6% to approximately 16.1%, reflecting a clear optimization trend. By 2022, the share of counties characterized by moderate unbalance and basic coordination decreased to 77.4%, whereas the proportion of moderately balanced and highly balanced coordination increased to 22.6% compared with 2012.

#### 3.2.2. Spatial Dimension Analysis

The ESDA method can analyze spatially correlated features in CCD. Firstly, we present the global Moran’s I for CCD (Table 4). All global Moran’s indices from 2012 to 2022 are greater than 0 and statistically significant at the 5% level; among them, only the values of 2021 and 2022 pass the significance test at the stricter 1% level, indicating a significant spatial agglomeration phenomenon. Furthermore, the indices show an upward trend, and all pass the significance test, suggesting that CCD exerts a strong influence on neighboring regions through spatial spillover effects during the study period.

**Figure 4:**
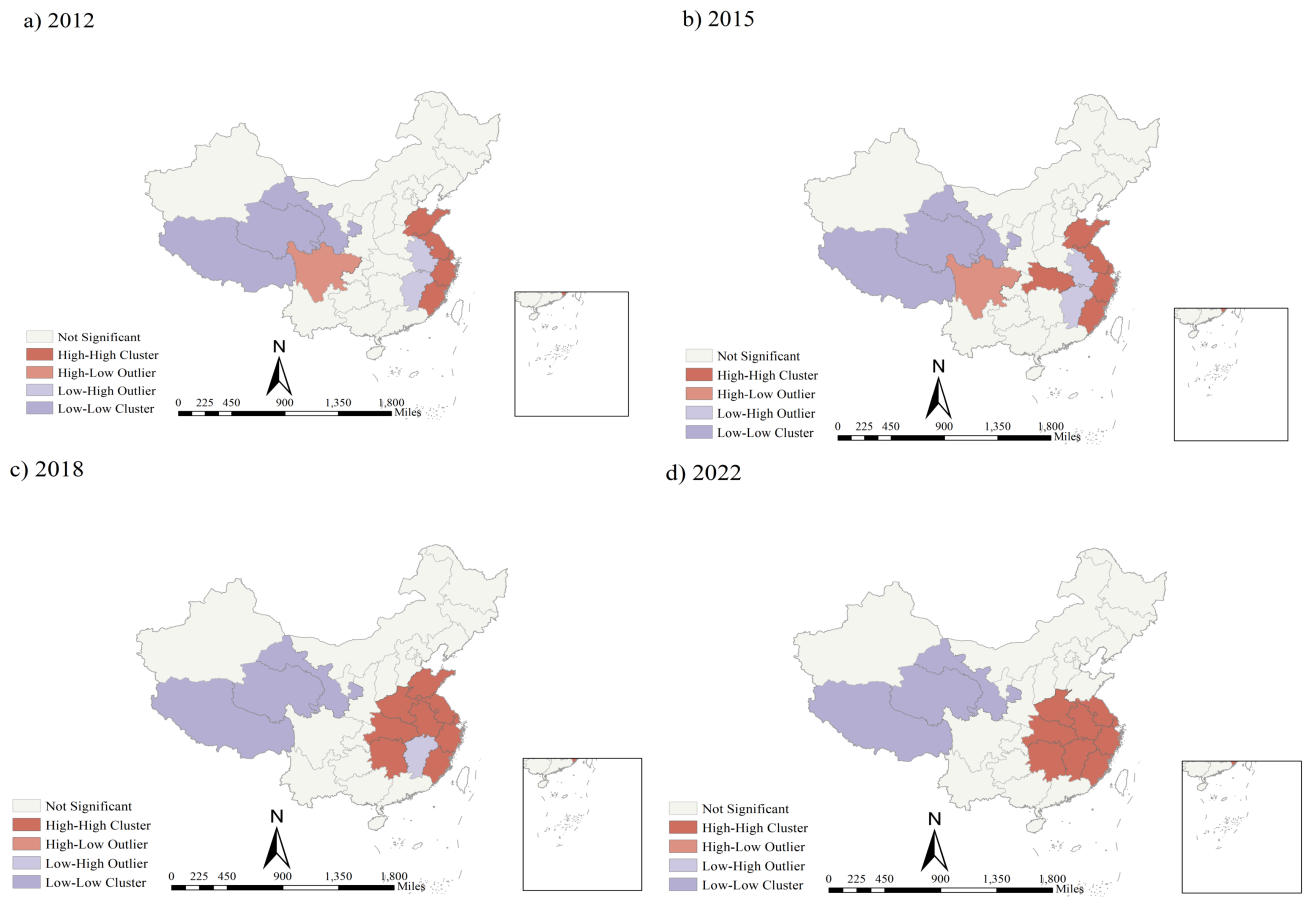
LISA cluster map of the CCD in China in selected years. (**a**) 2012; (**b**) 2015; (**c**) 2018; (**d**) 2022.

The spatial distribution of CCD values was analyzed at four cutoff time points: 2012, 2015, 2018, and 2022 (Figure 4). Combined with the LISA cluster map, the local agglomeration characteristics of CCD are further identified. The results show that the high-high type is mainly concentrated in the eastern region, particularly along the eastern coastal areas, where the synergy between subsystems is strong. In contrast, the low-low type provinces are mainly distributed in the western region, where limited medical resources and relatively underdeveloped economies contribute to weaker subsystem coordination. In addition, the number of high-high type provinces has increased slightly. Initially, the high-high type was scattered in five provinces: Fujian, Zhejiang, Shanghai, Jiangsu, and Shandong. By 2022, this cluster had expanded into a coherent “Yangtze River Delta-Middle Reaches of the Yangtze River” coastal-riparian corridor (with the addition of Jiangxi, Hunan, Hubei, Henan, and Anhui). This spatial expansion is consistent with the hypothesis that the eastern coastal provinces may exert spillover effects on neighboring regions.

However, no new high-value clusters have been formed in the central and western regions, and the potential for regional synergy has not been fully released. Meanwhile, the low-low regions have remained stable in Qinghai, Gansu, and their surrounding provinces, with almost unchanged scope and quantity, forming a long-lasting and stable “western depression”.

### 3.3. Levels and Sources of Spatial-Temporal Differences in CCD

**Figure 5:**
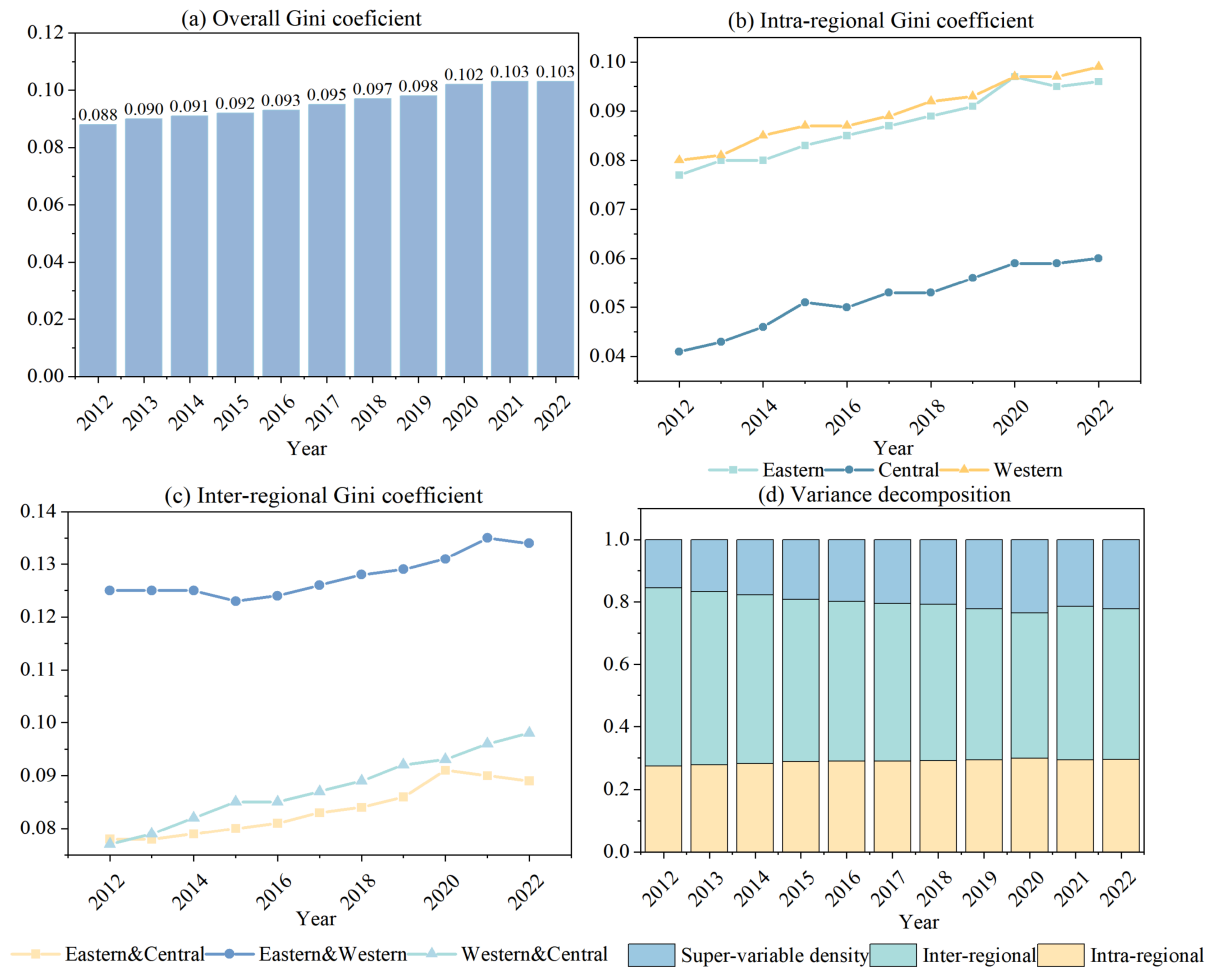
Dagum Gini coefficient decomposition. (**a**) overall Gini coefficient; (**b**) intra-regional Gini coefficient; (**c**) inter-regional Gini coefficient; (**d**) variance decomposition.

The study further explores the root causes of the differences as well as the trend of the overall differences. Figure 5a explores the root causes of these differences and the trend of overall disparities. The overall Gini coefficient rose steadily from 0.088 in 2012 to 0.103 in 2022, at an average annual growth rate of approximately 1.59%. At the intra-regional level (Figure 5b), the Gini coefficients of the three regions all showed an upward trend: the Eastern region rose from 0.077 to 0.096, the Central region from 0.041 to 0.060, and the Western region from 0.080 to 0.099.

The inter-regional Gini coefficients followed the order of Eastern-Western > Western-Central > Eastern-Central throughout the period. Specifically, the Eastern-Western coefficient increased from 0.125 in 2012 to 0.134 in 2022, the Western-Central coefficient rose from 0.077 to 0.098, and the Eastern-Central coefficient grew from 0.078 to 0.089 (Figure 5c). From the perspective of the sources and contribution rates of regional differences in the MRR system, inter-regional differences were the primary source of spatial disparities in the CCD, with an average contribution rate of 51.11%, which fluctuated downward from 57.06% in 2012 to 48.25% in 2022. The average contribution rate of intra-regional differences was 28.90%, showing a slight upward trend from 27.47% to 29.58%. The average contribution rate of the super-variable density component was 19.99%, increasing from 15.47% to 22.17%, and it remained the smallest contributor overall (Figure 5d).

### 3.4. Factor Explanatory Power of CCD

This study used CCD as the response variable and eight external variables as predictors: average years of education (AYE), government health expenditure (GHE), R&D funding (R&D), population density (PD), ageing level (AL), road network density (RND), internet broadband access ports (IBAP), and PM2.5 concentration (PM2.5). These predictors were not used in the construction of the CCD index. Their definitions and calculation methods are shown in Table 5. Variance inflation factors (VIFs) were calculated to assess multicollinearity among the external predictors. All VIF values ranged from 1.346 to 3.809, remaining below both the commonly used threshold of 10 and the stricter reference value of 5, indicating no severe multicollinearity among the retained predictors (Table S3).

**Figure 6:**
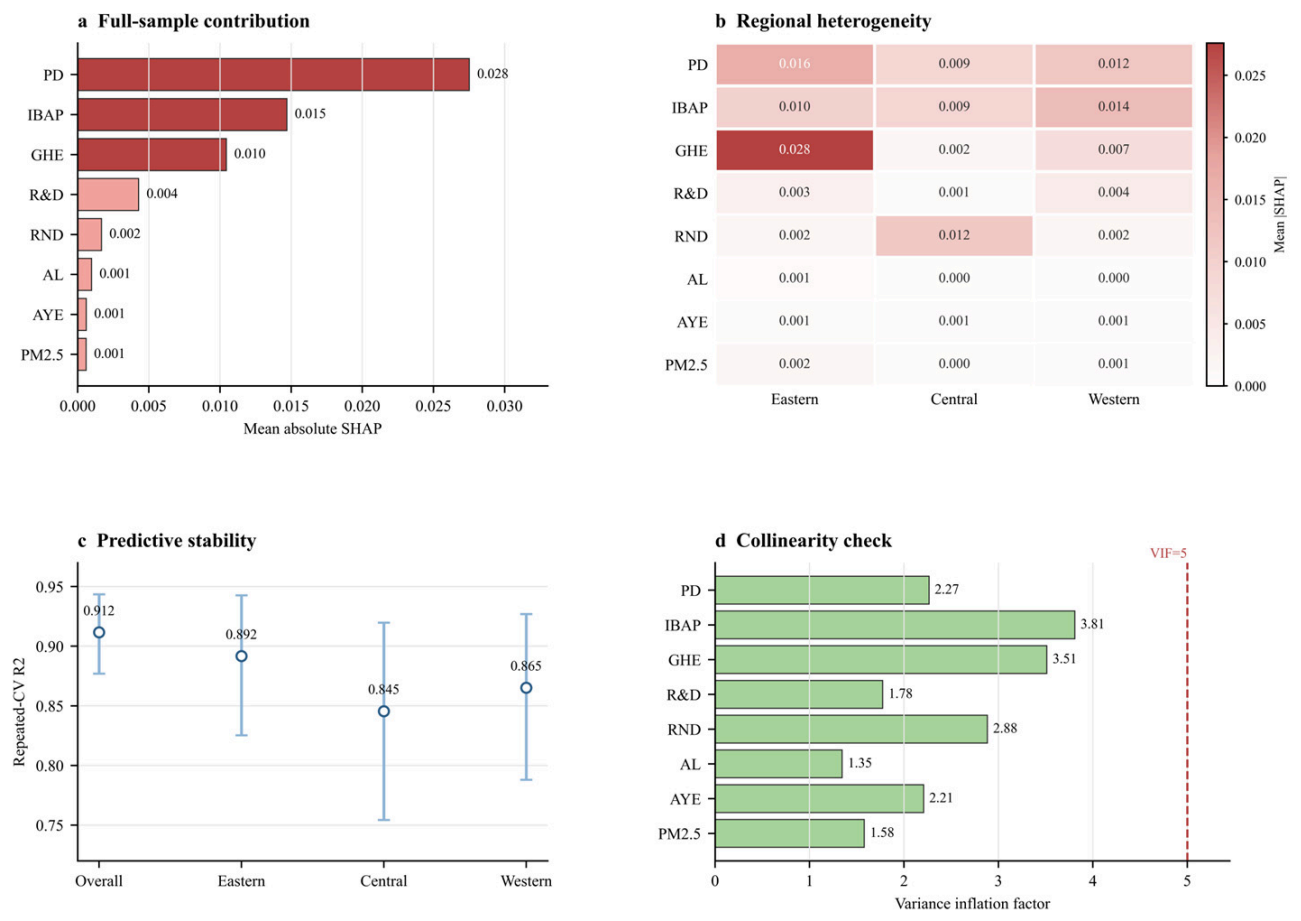
SHAP contribution ranking of external factors. (**a**) Full-sample contribution; (**b**) regional heterogeneity; (**c**) predictive stability; (**d**) collinearity check.

After confirming the absence of severe multicollinearity, an XGBoost model was fitted to evaluate the external predictive contributors associated with CCD. Repeated five-fold cross-validation showed stable predictive performance. In the full sample, the mean R2 was 0.912, with an RMSE of 0.019 and an MAE of 0.014. Region-specific models also showed reasonable performance, with R^2^ values of 0.892 for eastern China, 0.846 for central China, and 0.865 for western China. The corresponding resampling intervals are reported in Table S4. SHAP analysis showed that PD was the leading external predictive contributor in the full sample, with a mean absolute SHAP value of 0.028, followed by IBAP (0.015) and GHE (0.010). The regional results revealed clear heterogeneity. In the eastern region, GHE ranked first, followed by PD and IBAP. In the central region, RND made the largest contribution, followed by IBAP and PD. In the western region, IBAP ranked first, followed by PD and GHE. These results indicate that the external predictors associated with CCD differ across regions (Figure 6 & Table S5).

## 4. Discussion

In terms of research perspective, medical service capacity, residents’ health level, and regional economic development have become the focus of numerous studies. However, previous studies have rarely explored the interactive relationships among these subsystems [42,43]. For instance, Xu [44] examined the coupling coordination between health resource allocation and health service utilization using a two-subsystem model, which does not explicitly incorporate residents’ health outcomes or regional economic development as independent subsystems. In contrast, this study is grounded in a holistic systematic perspective and focuses on examining the synergistic effects among MRR. Compared with simply analyzing one-way impacts, this research perspective can more comprehensively capture complex.

At the sub-system level, from 2012 to 2022, the composite index of the MRR system in China grew slowly, with an annual growth rate of merely 0.54%. Such modest progress mainly resulted from uncoordinated development across the three subsystems. Medical service capacity and regional economic development expanded rapidly by 2.86% and 2.55% per year, whereas residents’ health levels stayed at a high level, thus imposing a ceiling on overall improvement. The expansion of medical service capacity was supported by sustained investment under policies such as the Healthy China 2030 Planning Outline and national health system reform, which broadened medical resource supply, upgraded facilities and technologies, and strengthened system efficiency and accessibility [45]. However, growth slowed in later years due to diminishing marginal returns and temporary disruptions from the pandemic. Meanwhile, regional economic development improved consistently but remained highly unequal across provinces, restricting the balanced distribution of medical resources. Supported by long-term public health investment, residents’ health was no longer the main constraint on coordinated development.

Analyses of the CCD reveal that the MRR system has experienced a steady increase over recent years, with a significant positive spatial correlation throughout the study period, as the consistently positive global Moran’s I index confirms that the spatial distribution of China’s MRR system is not random but exhibits clear spatial regularity—geographically adjacent regions tend to display similar levels of development across the three subsystems. These findings are consistent with those of similar studies [46]. The divergent trajectories of high-high (H-H) and low-low (L-L) clusters are shaped by two opposing spatial mechanisms: the expansion of H-H clusters in eastern China is driven by the spillover effect of the Yangtze River Delta, where its advanced healthcare resources, economic vitality, and integrated health policies have radiated to adjacent central provinces, breaking the initial pattern of scattered high-value areas and forming a continuous synergy corridor; the persistent L-L clusters in western China reflect a stable low-level equilibrium trap, where economic underdevelopment, insufficient medical investment, and poor health outcomes mutually reinforce one another, rendering the region resistant to weak spatial spillovers from the east. This imbalance highlights the limited reach of natural spillover effects and underscores the need for targeted policy interventions to break the coordination bottleneck in western China.

The SHAP results indicate that PD had the largest model-based contribution to CCD prediction in the full sample. This finding is consistent with previous studies on health-economy coupling coordination. Higher population density may be associated with stronger agglomeration effects, broader medical service coverage, and more efficient health-resource allocation through economies of scale. Similar relationships have been reported in studies of health production efficiency, new urbanization, and provincial health-resource coordination in China [47]. The regional SHAP rankings further suggest that the external predictors associated with CCD vary across development contexts. In the eastern region, GHE ranked first among the predictors. This pattern is consistent with the role of public health expenditure associated with higher levels of medical infrastructure, health professional teams, and public health programs in fiscally stronger provinces [48]. In the central region, RND ranked first, indicating that transport accessibility may be particularly relevant in provinces undergoing industrial and urban transition. Improved road-network density can reduce transaction costs and facilitate the movement of capital, labor, patients, and health services [49]. In the western region, IBAP emerged as the leading predictor, suggesting that digital infrastructure may be associated with greater use of telemedicine and health-information exchange, potentially mitigating geographic barriers [50].

## 5. Limitations

This study has several limitations. First, the findings are based on provincial-level data in China, which may limit their generalizability to other countries with different health systems, economic structures, and social contexts. Second, some unobserved confounding factors are not fully considered in the model. Future studies could use multi-scale data, longer time series, and cross-country comparisons to improve the robustness and generalizability of the results. Third, from a methodological perspective, entropy weighting determines weights merely by data dispersion and neglects practical connotations of indicators; applied machine learning is subject to black-box constraints and potential overfitting risks, hindering result interpretation and out-of-sample promotion.

## 6. Conclusions

This study clarifies the spatiotemporal evolution characteristics and important contributors of the CCD of the MRR composite system in China from 2012 to 2022. It confirms the slow upward trend of overall CCD, obvious regional disparities, significant spatial agglomeration effects, and heterogeneous important contributors across different regions. These findings provide descriptive evidence that may inform future policy discussions.

## Figures and Tables

**Table 1:** MRR composes evaluation index system construction.

Dimensions	Sub-Subsystems	Indicators	Symbol	Weight	Effect Direction
Medical service capacity	B1 Service input	C1 Number of health institutions per 1000 people	NI	0.142	Positive
		C2 Number of beds per 1000 people	NB	0.071	Positive
		C3 Number of health personnel per 1000 people.	NH	0.097	Positive
	B2 Service output	C4 Number of outpatient visits	OV	0.250	Positive
		C5 Number of inpatient visits	IV	0.208	Positive
		C6 Average daily number of patient visits. per doctor	AD	0.177	Positive
	B3 Service efficiency	C7 Average length of hospital stays	HS	0.044	Negative
		C8 Bed utilization rate	BU	0.010	Positive
Residents’ health level	B4 National health level	C9 Average life expectancy	LE	0.196	Positive
		C10 Infectious disease incidence	ID	0.368	Negative
	B5 Maternal mortality index	C11 Maternal mortality rate	MMR	0.328	Negative
	B6 Perinatal mortality index	C12 Perinatal mortality rate	PMR	0.108	Negative
Regional economic development	B7 Economic structure	C13 Share of primary industry in GDP	PI	0.091	Positive
		C14 Share of secondary industry in GDP	SI	0.037	Positive
		C15 Share of tertiary industry in GDP	TS	0.070	Positive
	B7 Economic vitality	C16 Consumption rate	CR	0.045	Positive
		C17 GDP growth rate	GGR	0.012	Positive
		C18 Foreign trade dependence	FTD	0.237	Positive
	B8 Economic scale	C19 GDP	GDP	0.175	Positive
		C20 Fixed-asset investment	FAI	0.174	Positive
		C21 Government budget revenue	GBR	0.158	Positive

**Table 2:** Robustness test of coupling coordination results under different weighting schemes.

Scheme	Medical Service	Residents’ Health	Regional Economic	Correlation
	Capacity	Level	Development	Coefficient
Original	1/3	1/3	1/3	1
Scheme 1	0.30	0.40	0.30	0.997
Scheme 2	0.40	0.25	0.35	0.997

**Table 3:** Classification of coupling coordination levels for CCD.

Level	Degree
[0.00–0.30]	Seriously unbalanced
(0.30–0.40]	Moderately unbalanced
(0.40–0.50]	Basically balanced
(0.50–0.60]	Moderately balanced
(0.60–1.00]	Highly balanced

**Table 4:** Global Moran’s index.

Year	I	Z-Value	*p*-Value	Result
2012	0.1297	2.1660	0.0303	Clustering
2013	0.1298	2.1657	0.0303	Clustering
2014	0.1400	2.2995	0.0215	Clustering
2015	0.1527	2.4689	0.0136	Clustering
2016	0.1504	2.4376	0.0148	Clustering
2017	0.1536	2.4758	0.0133	Clustering
2018	0.1518	2.4517	0.0142	Clustering
2019	0.1547	2.4873	0.0129	Clustering
2020	0.1499	2.4212	0.0155	Clustering
2021	0.1756	2.7620	0.0057	Clustering
2022	0.1719	2.7131	0.0067	Clustering

**Table 5:** External predictors are associated with CCD.

External Associated Predictors	Calculation and Description of the Indicators	Symbol	References
Average years of education	(Number of individuals with primary education × 6 + Number of individuals with junior secondary education × 9 + Number of individuals with senior secondary and vocational education × 12 + Number of individuals with associate degrees and above × 16)/Total population aged 6 and above.	AYE	[33]
Government health expenditure	Reflects government support.	GHE	[34]
R&D funding	Reflects technology and innovation.	R&D	[35]
Population density	Total population of the region/Land area of the region.	PD	[36]
Aging level	Population aged 65 and over/Total Population of the region.	AL	[37]
Road network density	Total length of regional highways/Regional land area.	RND	[38]
Internet broadband access ports	The total number of internet broadband access ports/The total population of the region.	IBAP	[39]
PM2.5 concentration	Arithmetic average of the annual average concentration of PM2.5 (μg/m^3^) at each monitoring point in the region.	PM2.5	[40,41]

## Data Availability

The data analyzed in this study were derived from publicly available official statistical yearbooks (e.g., China Statistical Yearbook) accessed via the CNKI China Economic and Social Big Data Research Platform, subject to the terms and conditions of the CNKI platform.

## Supplementary Materials

The following supporting information can be downloaded at: https://www.mdpi.com/article/10.3390/healthcare14152241/s1. Table S1: The CCD measurement results under scheme1; Table S2: The CCD measurement results under scheme 2; Table S3: Multicollinearity test of variables; Table S4: Performance of the external factor model via repeated cross-validation; Table S5: Top Three External Factors Ranked by SHAP Contribution Across Samples.

## Abbreviations

The following abbreviations are used in this manuscript:

CCD	Coupling Coordination Degree
CCDM	Coupling Coordination Degree Model
MRR	Medical service capacity, Residents’ health level and Regional economic development composite systems.
