# Supplementary material for: Coupling Coordination Analysis of Medical Service Capacity, Residents’ Health Level and Regional Economic Development in China
Source: Healthcare (Basel). 2026 Jul 23;14(15):2241. doi: 10.3390/healthcare14152241 (PMC13466204; doi:10.3390/healthcare14152241)
Supplement: Supplementary file 1 [file healthcare-14-02241-s001.zip › healthcare-4291746-supplementary.pdf]

Table S1. The CCD measurement results under scheme1 ($\alpha = 0.30, \beta = 0.40, \Upsilon = 0.30$).

Number	2012	2013	2014	2015	2016	2017	2018	2019	2020	2021	2022
1	0.555	0.572	0.589	0.600	0.611	0.625	0.637	0.649	0.618	0.624	0.620
2	0.712	0.717	0.711	0.699	0.699	0.699	0.705	0.713	0.676	0.705	0.704
3	0.610	0.616	0.619	0.621	0.624	0.629	0.636	0.644	0.632	0.648	0.651
4	0.513	0.525	0.534	0.539	0.546	0.539	0.544	0.550	0.521	0.519	0.500
5	0.760	0.772	0.780	0.792	0.801	0.807	0.816	0.831	0.806	0.832	0.825
6	0.560	0.572	0.586	0.595	0.605	0.612	0.622	0.633	0.619	0.630	0.627
7	0.491	0.505	0.518	0.539	0.553	0.569	0.582	0.588	0.583	0.596	0.594
8	0.512	0.514	0.517	0.518	0.523	0.524	0.529	0.534	0.536	0.553	0.551
9	0.589	0.596	0.622	0.630	0.636	0.643	0.658	0.673	0.669	0.655	0.650
10	0.597	0.617	0.632	0.645	0.658	0.674	0.693	0.709	0.697	0.711	0.710
11	0.540	0.555	0.556	0.553	0.561	0.573	0.577	0.590	0.541	0.546	0.534
12	0.589	0.602	0.605	0.638	0.627	0.637	0.646	0.656	0.604	0.639	0.643
13	0.560	0.574	0.589	0.601	0.610	0.617	0.626	0.638	0.621	0.624	0.618
14	0.536	0.548	0.542	0.554	0.562	0.549	0.565	0.561	0.546	0.552	0.534
15	0.678	0.692	0.708	0.718	0.725	0.736	0.745	0.756	0.749	0.762	0.765
16	0.549	0.556	0.568	0.574	0.583	0.593	0.603	0.616	0.607	0.623	0.624
17	0.593	0.614	0.615	0.600	0.593	0.598	0.596	0.599	0.561	0.562	0.549
18	0.522	0.534	0.546	0.552	0.557	0.563	0.564	0.573	0.542	0.544	0.534
19	0.500	0.510	0.519	0.515	0.517	0.526	0.517	0.518	0.502	0.502	0.497
20	0.456	0.461	0.473	0.476	0.479	0.484	0.489	0.499	0.488	0.485	0.468
21	0.653	0.678	0.686	0.697	0.704	0.711	0.724	0.720	0.718	0.741	0.739
22	0.523	0.539	0.546	0.557	0.570	0.566	0.577	0.576	0.556	0.565	0.549
23	0.551	0.568	0.580	0.588	0.599	0.612	0.623	0.631	0.608	0.615	0.610
24	0.703	0.706	0.714	0.719	0.723	0.726	0.728	0.731	0.708	0.741	0.715
25	0.607	0.622	0.635	0.644	0.655	0.670	0.683	0.696	0.662	0.667	0.655
26	0.599	0.611	0.621	0.616	0.619	0.620	0.613	0.614	0.578	0.598	0.583
27	0.522	0.528	0.527	0.521	0.523	0.527	0.529	0.528	0.510	0.511	0.498
28	0.555	0.554	0.560	0.567	0.576	0.577	0.570	0.588	0.561	0.571	0.561
29	0.537	0.549	0.562	0.568	0.578	0.590	0.609	0.621	0.589	0.589	0.580
30	0.684	0.696	0.708	0.715	0.724	0.738	0.749	0.762	0.737	0.768	0.772
31	0.547	0.561	0.574	0.576	0.579	0.583	0.588	0.597	0.588	0.600	0.596

Table S2. The CCD measurement results under scheme1 ($\alpha = 0.40, \beta = 0.25, \Upsilon = 0.35$)

Number	2012	2013	2014	2015	2016	2017	2018	2019	2020	2021	2022
1	0.553	0.569	0.587	0.598	0.608	0.622	0.634	0.646	0.615	0.621	0.617
2	0.709	0.713	0.708	0.696	0.695	0.696	0.701	0.710	0.672	0.702	0.701
3	0.607	0.613	0.616	0.618	0.621	0.626	0.633	0.641	0.629	0.645	0.648
4	0.511	0.522	0.531	0.536	0.544	0.536	0.541	0.548	0.518	0.516	0.498
5	0.757	0.769	0.776	0.789	0.797	0.803	0.812	0.827	0.802	0.829	0.821
6	0.558	0.569	0.583	0.592	0.602	0.609	0.619	0.630	0.616	0.627	0.624
7	0.489	0.503	0.515	0.536	0.550	0.566	0.579	0.585	0.580	0.593	0.592
8	0.509	0.511	0.515	0.516	0.520	0.522	0.526	0.531	0.533	0.550	0.548
9	0.587	0.593	0.619	0.627	0.633	0.640	0.655	0.670	0.666	0.652	0.647
10	0.594	0.614	0.629	0.642	0.655	0.671	0.690	0.705	0.694	0.708	0.707
11	0.537	0.553	0.553	0.550	0.559	0.570	0.574	0.587	0.538	0.543	0.531
12	0.586	0.599	0.602	0.635	0.624	0.634	0.643	0.653	0.601	0.636	0.640
13	0.557	0.572	0.586	0.598	0.607	0.615	0.623	0.635	0.618	0.621	0.615
14	0.533	0.546	0.539	0.552	0.560	0.546	0.562	0.559	0.543	0.549	0.531
15	0.675	0.689	0.705	0.714	0.721	0.733	0.742	0.752	0.746	0.759	0.761
16	0.547	0.553	0.565	0.571	0.580	0.591	0.600	0.613	0.604	0.620	0.621
17	0.590	0.611	0.612	0.597	0.590	0.596	0.593	0.596	0.559	0.559	0.546
18	0.519	0.531	0.544	0.549	0.554	0.560	0.561	0.570	0.540	0.541	0.531
19	0.498	0.507	0.516	0.512	0.515	0.523	0.515	0.515	0.500	0.500	0.494
20	0.454	0.459	0.471	0.473	0.477	0.481	0.487	0.497	0.485	0.483	0.466
21	0.649	0.674	0.683	0.694	0.701	0.708	0.720	0.717	0.714	0.737	0.735
22	0.520	0.537	0.543	0.555	0.567	0.563	0.574	0.574	0.553	0.562	0.546
23	0.548	0.565	0.577	0.585	0.596	0.609	0.620	0.628	0.606	0.612	0.608
24	0.699	0.702	0.710	0.715	0.720	0.723	0.725	0.728	0.705	0.737	0.712
25	0.604	0.619	0.632	0.641	0.652	0.667	0.680	0.693	0.658	0.664	0.652
26	0.597	0.608	0.618	0.613	0.616	0.617	0.610	0.611	0.575	0.595	0.580
27	0.520	0.526	0.524	0.519	0.520	0.524	0.527	0.526	0.508	0.509	0.496
28	0.553	0.552	0.558	0.564	0.573	0.575	0.568	0.585	0.559	0.568	0.559
29	0.535	0.546	0.560	0.565	0.575	0.587	0.606	0.618	0.587	0.587	0.577
30	0.680	0.693	0.705	0.712	0.721	0.735	0.745	0.758	0.734	0.764	0.769
31	0.544	0.559	0.571	0.573	0.576	0.581	0.585	0.594	0.585	0.597	0.594

Table S3. Multicollinearity test of variables.

Variable	Variance inflation factor (VIF)
IBAP	3.809
GHE	3.513
RND	2.884
PD	2.267
AYE	2.208
R&D	1.775
PM2.5	1.58
AL	1.346

Table S4. Performance of the external factor model via repeated cross-validation.

Region	R^2 (2.5%-97.5%)	RMSE (2.5%-97.5%)	MAE (2.5%-97.5%)
Overall	0.912 [0.877-0.944]	0.019 [0.015-0.023]	0.014 [0.011-0.018]
Eastern	0.892 [0.825-0.943]	0.020 [0.014-0.028]	0.016 [0.011-0.022]
Central	0.846 [0.754-0.920]	0.015 [0.009-0.023]	0.011 [0.007-0.017]
Western	0.865 [0.788-0.927]	0.015 [0.010-0.022]	0.012 [0.008-0.016]

Table S5. Top Three External Factors Ranked by SHAP Contribution Across Samples.

Sample	Rank	Variable	Mean Absolute SHAP Value	Bootstrap 95% Confidence Interval	Number of Observations
Overall	1	PD	0.028	[0.026,0.029]	341
Overall	2	IBAP	0.015	[0.014,0.016]	341
Overall	3	GHE	0.010	[0.010,0.011]	341
Eastern region	1	GHE	0.028	[0.026, 0.030]	121
Eastern region	2	PD	0.016	[0.015, 0.018]	121
Eastern region	3	IBAP	0.010	[0.009, 0.011]	121
Central region	1	RND	0.012	[0.011, 0.012]	88
Central region	2	IBAP	0.009	[0.008, 0.010]	88
Central region	3	PD	0.009	[0.008, 0.010]	88
Western region	1	IBAP	0.014	[0.012,0.015]	132
Western region	2	PD	0.012	[0.011,0.012]	132
Western region	3	GHE	0.007	[0.007,0.008]	132
